# Do people with risky behaviours participate in biomedical cohort studies?

**DOI:** 10.1186/1471-2458-6-11

**Published:** 2006-01-23

**Authors:** Anne W Taylor, Eleonora Dal Grande, Tiffany Gill, Catherine R Chittleborough, David H Wilson, Robert J Adams, Janet F Grant, Patrick Phillips, Richard E Ruffin, the North West Adelaide Health Study Team

**Affiliations:** 1 Population Research & Outcome Studies Unit, South Australian Department of Health, Australia; 2 Health Observatory, Department of Medicine, The University of Adelaide, Australia; 3 Department of Medicine, The University of Adelaide, South Australia, Australia; 4 Department of Endocrine Services Queen Elizabeth Hospital, Woodville South Australia, Australia

## Abstract

**Background:**

Analysis was undertaken on data from randomly selected participants of a bio-medical cohort study to assess representativeness. The research hypotheses was that there was no difference in participation and non-participations in terms of health-related indicators (smoking, alcohol use, body mass index, physical activity, blood pressure and cholesterol readings and overall health status) and selected socio-demographics (age, sex, area of residence, education level, marital status and work status).

**Methods:**

Randomly selected adults were recruited into a bio-medical representative cohort study based in the north western suburbs of the capital of South Australia – Adealide. Comparison data was obtained from cross-sectional surveys of randomly selected adults in the same age range and in the same region. The cohort participants were 4060 randomly selected adults (18+ years).

**Results:**

There were no major differences between study participants and the comparison population in terms of current smoking status, body mass index, physical activity, overall health status and proportions with current high blood pressure and cholesterol readings. Significantly more people who reported a medium to very high alcohol risk participated in the study. There were some demographic differences with study participants more likely to be in the middle level of household income and education level.

**Conclusion:**

People with risky behaviours participated in this health study in the same proportions as people without these risk factors.

## Background

Numerous large and important cohort studies have been established overseas [1-6] and in Australia [7-9]. Many of the cohort studies undertaken are based on volunteers or clinical/convenience samples and the follow-up is based on self-report data or record linkages. The establishment of population-based, biomedical, cohort studies using a random sample is less common [1,2,5,9].

Cohort studies are based on the assessment of an individual at several points in time and, by recalling or re-contacting each individual, assessing the process and transition of the individual along the disease and life-course continuum [10,11]. As argued by Szklo [12] there are numerous, and obvious, advantages if population-based cohort studies are representative of their defined population. The translations of the data into population estimates over time enable casual relationships to be explored and the ability to separate out the effects of age and maturation, although the main aims are to undertake intra-group comparisons and follow changes over time [12,13]. Increasing emphasis is being placed upon these longitudinal data to inform policy makers, health promoters and health planners. With the ageing of the population and the resultant cost pressures placed upon health systems, these data are also being used to make informed predictions about the future use of health services, mortality and morbidity patterns. Cohort studies can provide unique data that provides a more detailed understanding of complex health issues, providing life-course analytical and useful evaluation research opportunities.

Biomedical cohort studies are very costly and logistically difficult to administer [12,14]. The benefits and uses of the data are compromised if bias exists. A high initial response rate, a representative initial sample and a low attrition rate are areas where effort needs to be invested to limit selection bias [15-17]. While loss to follow-up is a somewhat expected consequence of the longitudinal nature of all cohort studies, the representativeness of the initial sample, and subsequent ongoing continual assessment of representativeness, are important aspects that warrant investigation [11-13].

This paper investigates the representativeness of an initial cohort to determine if people undertaking risky behaviours were less likely to participate in a major biomedical cohort study and to study the direction and magnitude of any bias found. As argued by Grimes [11], cohort studies should be upfront in identifying and describing the potential effects of any bias and assess similarities and dissimilarities of respondents. This paper aims to identify and describe these biases for a major cohort study established in the western and northern suburbs of Adelaide, the capital of South Australia in which over 4000 randomly selected adults have been recruited. The overall aim of this cohort study is to follow the continuum of selected chronic diseases and associated risk factors.

## Methods

The North West Adelaide Health Cohort Study (NWAHCS) recruited between 2000 and 2002 with a total of n = 4060 adults participating. Randomly selected telephone numbers listed in the relevant postcodes (that equated to the boundaries of the suburbs selected to be included in the study) were drawn from the most current Electronic White Pages. A letter of invitation to participate was sent to these households followed within 10 days by a telephone call from trained health study recruiters. A randomly selected adult within the household (those with the next birthday aged 18 years and older) was asked to participate in the study. At each appointment, the participant was given additional detailed information about the study and asked to sign consent forms for participation in the study. The information given highlighted the longitudinal nature of the study, and participants were informed that they may be invited to participate in health-related sub-studies. Prior to the study commencing, approval for the research was obtained from the North West Adelaide Health Service Ethics of Human Research Committee.

Appointments were made for participants in one of the two hospital-based clinics in the region and participants were sent an information folder that included a questionnaire with questions on chronic disease, alcohol consumption, physical activity levels, quality of life and socio-economic details (including highest education level, marital status, work status, country of birth and household income level). Age, sex, smoking status, height, weight, and ever being told they had high blood pressure or high cholesterol were asked in the recruitment telephone interview. At the clinic a range of assessments were made including taking blood (to test fasting plasma glucose, lipids, HbA1c), skin prick tests to common allergens and spirometry lung function tests.

The overall response rate of the completed telephone interview, self-completed questionnaire and clinic biomedical assessment (including blood sample) was 49.6% (69% of those interviewed). This paper assesses data associated with the respondents who completed all aspects of the study. Full details of the methodology have been previously published [18-20].

To examine the representiveness of the NWAHS sample with regard to age, sex, area of residence and socio-economic status, a comparison was made using Australian Bureau of Statistics (ABS) Census figures. Socio-economic status was measured using the Socio Index for Areas, Index of Relative Social Disadvantage (SEIFA IRSD) [21].

To compare the other demographics and social characteristics of the respondents and the population estimates of key health risk factors, a comparison against a population-based survey, the South Australian Surveillance and Monitoring System (SAMSS), was undertaken. SAMSS is a representative, on-going, population household telephone interview surveillance/survey of the South Australian population based on EWP sampling and has operated each month since July 2002 using a consistent methodology [22]. This involves a random sample of SA households with one person selected at random in each household according to next birthday. Trained health interviewers interview respondents using computer assisted telephone interviewing (CATI) technology and there is no replacement for non-respondents. From July 2002 to June 2004, n = 2904 adults in the NW suburbs of Adelaide were interviewed providing a non-replacement response rate of 68.7%. To compare physical activity rates, data from the South Australian Health Monitor were used. Methodology of this CATI survey, operated three times a year, is similar to SAMSS and has been detailed elsewhere [23]. This is a separate comparable survey with a separate sample.

While the questions asked in NWAHCS and SAMSS were identical for age, sex, country of birth, household income, alcohol consumption, height and weight (to calculate body mass index (BMI)), current high blood pressure, current high cholesterol, physical activity and self-reported health status there were slight differences in wording of the question for highest education level, marital status, work status variables and smoking status. Questions on height, weight, blood pressure and cholesterol were only asked of the second half of the respondents although measurements in the clinic were undertaken on all participants.

All analyses were limited to data on respondents aged 18 years and over in the same geographical area to correspond to the NWAHCS sample. Data were weighted by age, sex, region and probalility of selection within the household to the 2001 ABS Census data for SA to provide estimates that were representative of the region's population. The comparison for age and sex using the ABS data used both weighted and un-weighted data. Significance was tested using SPSS V12.0 and EpiInfo Version 6 *X*^2 ^tests with a 0.05 level of significance. Adjusted standardized residuals were obtained using the methods of Haberman [24] and were used to test deviations from expected values separately in each cell. Bonferroni corrections were applied for multiple testing.

## Results

Initial analysis using un-weighted data showed that significantly less younger people (< 40 years) and more older (40+ years) were recruited into the cohort study when compared to Census data. There were no differences by sex or area of residence (Table 1). Table 2 highlights the differences by SEIFA quintiles with study participants more likely to be in the 3^rd ^quintile and less likely to be in the 4^th ^quintile of relative socio-economic disadvantage.

Table 3 highlights other demographic comparisons. There were statistically significant differences by education level, with NWAHCS participants more likely to have trade, certificate or diploma qualifications and less likely to have just secondary school qualifications or to have undertaken tertiary study than participants in SAMSS. There were no statistically significant differences by marital status or work status. The NWAHCS had a statistically significant higher proportion of people born in the United Kingdom or Ireland and a lower proportion of Australian-born. There were also differences in the household income level groups with the NWAHCS participants more likely to be in the $40–80,000 bracket and less likely to be in the $80,000+ bracket.

Table 4 shows the significant differences between the study participants and the comparative population for health risk factors. There was no difference by smoking status, physical activity level, general health status or the proportion with current HBP or current high cholesterol. NWAHCS participants were more likely to be in the intermediate to very high alcohol risk category and less likely to be in the underweight category of BMI.

## Conclusion

This cohort study has offered a unique opportunity to study chronic disease and related risk factors and to define the relationship between lifestyle and health and disease in the Australian population. Cohort studies are one of the most important tools for epidemiological investigation but random sampling cohort studies are often marred by biased samples, low response rates and high loss to follow-up [12,17]. Erroneous conclusions can be made if confounding factors are not incorporated in analytical comparisons and models [25]. Bias can be corrected providing confounding was anticipated and confounding factors are appropriately controlled [26,27]. This analysis has highlighted the variables that need consideration in future analyses associated with the NWAHCS.

This analysis has shown that in terms of bias associated with risk factors, the cohort participants are not dramatically unlike the community they represent. Their overall self-reported health status is the same, there is the same proportion of current smokers, their overall BMI status (except for underweight) is similar and the same proportion had current high blood pressure or high cholesterol readings. The only major difference was in terms of alcohol consumption with the cohort participants more likely to consume alcohol at an intermediate to high risk level.

In terms of demographic and social characteristics there were no differences by marital status or work status. The un-weighted comparison showed that less younger people and more older persons were recruited into the study. NWAHCS participants were also more likely to be born in UK or Ireland and less likely to be Australian born. These demographic differences could be explained by the fact that NWAHCS participants knew they were being recruited into a bio-medical cohort study with an outlay of personal time and effort required. The clinical meaning associated with the bias associated with this recruitment means we are missing out on the younger persons (expected to be healthier) and gaining more older persons (expected to be unhealthy) although the weighting of the data would counteract some of this bias. The country of birth differences should not affect clinical results as Australian-born and those born in UK/Ireland have similar heritages and social indicators. All future analyses and assumptions will take into account these differences and the fact that the study participants were more likely to have middle levels of education and be more likely to be in the middle levels of income.

The strength of this study lies in its representative nature, the large random sample and the high response rate. Although the response rate associated with the complete study involvement, including obtaining blood and other bio-medical measurements, was 49.6% (69% of people interviewed by telephone), this is high when compared to other recent, comparable Australian studies. The *AusDiab *study recorded a response rate of 28% and a recent pilot for a national Australian biomedical study reported obtaining blood from 23% of their sample [28]. There is a trend towards lower response rates in all types of population surveys as people protect their privacy, are overwhelmed by marketing telephone calls or mail outs. The additional commitments associated with involvement in a cohort study add to respondent burden. To overcome some of the initial bias afforded to the response rate, the data were weighted by age and sex. The weights reflect unequal sample inclusion probability and compensate for differential non-response. Theoretically the weighted analyses should provide reliable population estimates of health phenomena.

To increase the initial response rate the study team implemented a range of well recognized survey techniques. These included consideration of timing of the initial phone contact, timing of phone call and technique (questionnaire length, size), training of recruitment staff, marketing, branding, and a free-call telephone number for inquiries [29]. Early in the recruitment stage, qualitative interviews were conducted with subjects unwilling to participate in the cohort study and the findings incorporated into further recruitment procedures.

Limitations to this analysis include the use of data collected using mixed modes with comparisons based on data collected by telephone and self completion. Bias is known to exist by method of collection, especially in regard to socially desirable responses [30]. An additional weakness of the study was the lack of data on non-responders. Although some data were collected on people who refused to participate in the biomedical components of the study, a comparison with people unable to be contacted (non-responders to the recruitment telephone call) was not possible due to data limitations on non-responders. The self-report nature of the data collection could also contain an element of bias and therefore be seen as a limitation. The use of biomedical data in addition to the self-reported data in this study (height, weight, and blood pressure and cholesterol measurements) will allow comparisons to be made between reported and measured variables. This analysis is planned.

Many studies have assessed the characteristics of participants and non-participants in population surveys and questionnaire-based cohort studies. Details on, in-depth analysis of, and subsequent publishing of the initial samples for cohort studies that have been initiated in the last decade, in which participants commit to clinic assessments, are few. This study has shown that for this population, people who have risk factors for ill-health were just as likely as others to participate. This is of relevance for researchers interested in establishing a bio-medical cohort study and offers positive encouragement that the huge financial and human resource costs are worthwhile.

## Competing interests

The author(s) declare that they have no competing interests.

## Authors' contributions

AWT conceived of the study, managed the study and drafted the manuscript

EDG participated in the study design performed statistical analysis and helped to draft the manuscript

TKG participated in the study design and management and helped to draft the manuscript

CRC participated in the study design and management and helped to draft the manuscript

DHW conceived of the study, assisted in the management of the study and helped to review the manuscript

RJA assisted with the overall management of the study and helped to review the manuscript

JFG participated in co-ordination of the study and helped to review the manuscript

PJP conceived of the study and helped to review the manuscript

RER conceived of the study and helped to draft the manuscript

All authors read and approved the final manuscript.

## Pre-publication history

The pre-publication history for this paper can be accessed here:



## Figures and Tables

**Table 1:** Age and sex comparison between 2001 Census and NWAHCS

	**ABS 2001 Census**		**NWAHCS**							
			**Unweighted**				**Weighted**			

	**n**	**%**	**n**	**%**	**Chi-square value**	**P value**	**n**	**%**	**Chi-square value**	**P value**

**Sex**					1.54	0.21			0.23	0.64
Male	159919	48.5	1930	47.5			1985	48.9		
Female	169695	51.5	2130	52.5			2075	51.1		
**Age group**					373.13	<0.001			5.14	0.40
18 to 29 years	70665	21.4	467	11.5*			899	21.4		
30 to 39 years	66747	20.3	676	16.7*			790	20.3		
40 to 49 years	61669	18.7	876	21.6*			785	18.7		
50 to 59 years	49003	14.9	795	19.6*			600	14.9		
60 to 69 years	35300	10.7	622	15.3*			448	10.7		
70 years and over	46230	14.0	624	15.4*			538	14.0		
**Area of residence**					2.32	0.13			1.69	0.19
Western Suburbs	182706	55.4	2299	56.6			2209	54.4		
Northern Suburbs	146908	44.6	1761	43.4			1851	45.6		

Total	329614	100.0	4060	100.0			4060	100.0		

* denotes the category was statistically significantly different compared with ABS Census 2001 using adjusted standardized residuals greater than 2.0 and less than -2.0 (χ^2 ^test)

**Table 2:** SEIFA Index of relative socio-economic disadvantage – comparison between 2001 Census and NWAHCS

	**ABS 2001 Census**		**NWAHCS weighted**		**Chi-square value**	**P value**
	**n**	**%**	**n**	**%**		
**2001 SEIFA Index of Relative Socio-economic Disadvantage – quintile**					**23.59**	**<0.001**
Lowest quintile	124457	37.8	1504	37.0		
2nd quintile	65806	20.0	842	20.7		
3rd quintile	88351	26.8	1174	28.9		*
4th quintile	43479	13.2	445	11.0		*
Highest quintile	7521	2.3	94	2.3		

Total	329614	100.0	4060	100.0		

Note: Quintiles are based on NW Adelaide population, 18+ years

* denotes the category was statistically significantly different compared with ABS Census 2001 using adjusted standardized residuals greater than 2.0 and less than -2.0 (χ^2 ^test)

**Table 3:** Demographic comparison between NWAHCS and other comparable survey

	**NWAHCS**		**SAMSS** ** (NW Adelaide Study area)**		**Chi-square value**	**P value**
	**n**	**%**	**n**	**%**		
**Highest education level obtained**	*Self completed*		*Telephone*		**287.97**	**<0.001**
Secondary	1751	45.3	1742	60.1		*
Trade / Apprenticeship / Certificate / Diploma	1641	42.4	659	22.7		*
Bachelor degree or higher	475	12.3	500	17.2		*

Total	3867	100.0	2901	100.0		

**Marital status**	*Self completed*		*Telephone*		1.76	0.62
Married or living with partner	2525	62.7	1830	63.1		
Separated/divorced	331	8.2	242	8.3		
Widowed	232	5.8	183	6.3		
Never married	940	23.3	647	22.3		

Total	4028	100.0	2903	100.0		

**Work status**	*Self completed*		*Telephone*		6.72	0.08
Employed	2266	56.5	1633	56.2		
Unemployed	173	4.3	128	4.4		
Student	223	5.6	124	4.3		
Home duties, retired, other	1349	33.6	1019	35.1		

Total	4011	100.0	2904	100.0		

**Country of birth**	*Self completed*		*Telephone*		**8.67**	**0.03**
Australia	2865	70.6	2114	72.8		*
UK or Ireland	645	15.9	389	13.4		*
Europe, The USSR & the Baltic States	332	8.2	249	8.6		
Asia, Other	217	5.4	153	5.3		

Total	4060	100.0	2904	100.0		

**Gross annual household income**	*Self completed*		*Telephone*		**16.24**	**<0.001**
Up to $40,000	1910	50.2	1239	49.4		
$40,001 – $80,000	1399	36.8	858	34.2		*
More than $80,000	492	13.0	414	16.5		*

Total	3802	100.0	2510	100.0		

* denotes the category was statistically significantly different compared with SAMSS using adjusted standardized residuals greater than 2.0 and less than -2.0 (χ^2 ^test)

**Table 4:** Risk factor comparison between NWAHCS and other comparable survey

	**NWAHCS**		**SAMSS** ** (NW Adelaide Study area)**			**P value**
	**n**	**%**	**n**	**%**		
**Smoking status**	*Telephone*		*Telephone*		0.07	0.79
Non or ex-smokers	3051	75.6	2202	75.9		
Current smoker	985	24.4	700	24.1		

Total	4036	100.0	2902	100.0		

**Alcohol risk**	*Self-complete*		*Telephone*		**15.71**	**<0.001**
Non drinkers, no risk	2148	53.4	1550	53.4		
Low risk	1630	40.5	1236	42.6		
Intermediate to very high risk	244	6.1	116	4.0		*

Total	4023	100.0	2902	100.0		

**BMI**						
**Self-reported**	*Telephone*		*Telephone*		**11.00**	**0.01**
Underweight <18.50	14	1.0	65	2.4		*
Normal 18.50–24.99	589	43.0	1143	41.6		
Overweight 25.00–29.99	505	36.8	964	35.1		
Obese 30.00+	262	19.1	573	20.9		

Total	1371	100.0	2746	100.0		

**Current high blood pressure**						
**Self-reported**	*Telephone*		*Telephone*		1.73	0.19
No/Don't know	1284	84.2	1292	85.8		
Current HBP	242	15.8	213	14.2		

Total	1525	100.0	1505	100.0		

**Current high cholesterol**						
**Self-reported**	*Telephone*		*Telephone*		3.11	0.08
No/Don't know	1337	87.7	1350	89.7		
Current	188	12.3	155	10.3		

Total	1525	100.0	1505	100.0		

**Physical activity**						
**Self-reported**	*Self-complete*		*Telephone*		7.19	0.06
Sedentary	1037	28.1	131	25.3		
Low exercise level	1346	36.5	214	41.3		
Moderate exercise level	891	24.1	129	24.8		
High exercise level	417	11.3	45	8.6		
					1.70	0.19
Sedentary	1037	28.1	131	25.3		
Undertakes low to high levels of exercise	2655	71.9	387	74.7		

Total	3691	100.0	519	100.0		

**Self-reported general health status**	*Self-complete*		*Telephone*		0.51	0.48
Excellent, very good or good	3314	82.0	2363	81.4		
Fair or poor	725	18.0	541	18.6		

Total	4040	100.0	2904	100.0		

* denotes the category was statistically significantly different compared with SAMSS using adjusted standardized residuals greater than 2.0 and less than -2.0 (χ^2 ^test)
